# Four-dimensional computational ultrasound imaging of brain hemodynamics

**DOI:** 10.1126/sciadv.adk7957

**Published:** 2024-01-17

**Authors:** Michael D. Brown, Bastian S. Generowicz, Stephanie Dijkhuizen, Sebastiaan K. E. Koekkoek, Christos Strydis, Johannes G. Bosch, Petros Arvanitis, Geert Springeling, Geert J. T. Leus, Chris I. De Zeeuw, Pieter Kruizinga

**Affiliations:** ^1^ Department of Neuroscience, CUBE, Erasmus MC, Rotterdam, Netherlands.; ^2^ Department of Medical Physics and Biomedical Engineering, University College London, London, UK.; ^3^ Department of Neuroscience, Erasmus MC, Rotterdam, Netherlands.; ^4^ Department of Quantum and Computer Engineering, TU Delft, Delft, Netherlands.; ^5^ Department of Cardiology, Thorax Biomedical Engineering, Erasmus MC, Rotterdam, Netherlands.; ^6^ Experimental Medical Instrumentation, Erasmus MC, Rotterdam, Netherlands.; ^7^ Signal Processing Systems, Department of Microelectronics, TU Delft, Delft, Netherlands.; ^8^ Netherlands Institute for Neuroscience, Royal Dutch Academy for Arts and Sciences, Amsterdam, Netherlands.

## Abstract

Four-dimensional ultrasound imaging of complex biological systems such as the brain is technically challenging because of the spatiotemporal sampling requirements. We present computational ultrasound imaging (cUSi), an imaging method that uses complex ultrasound fields that can be generated with simple hardware and a physical wave prediction model to alleviate the sampling constraints. cUSi allows for high-resolution four-dimensional imaging of brain hemodynamics in awake and anesthetized mice.

## Teaser

The use of random wavefields enables affordable 3D ultrasound imaging of blood flow.

## INTRODUCTION

The advent of ultrafast ultrasound (>5000 frames per second) imaging over the past 20 years, enabled by increased computational power and parallel receive electronics, has spurred the development of multiple imaging modes for biomedical ultrasound (*1*, *2*). The formation of complete images within a short (<1 ms) temporal window allows for accurate quantification of tissue, blood, and contrast agent motion. This facilitates measurement of tissue elasticity and arterial stiffness (*3*, *4*), superresolution via localization and tracking of individual microbubbles (*5*, *6*) and vastly enhanced imaging of blood flow over a wide field of view (*7*). The latter has resulted in the emergence of functional ultrasound imaging (fUS or fUSi) a neuroimaging technique, which is able to detect small changes in cerebral blood volume induced by neurovascular coupling (*8*, *9*). In comparison to other neuroimaging modalities such as functional magnetic resonance imaging, fUS offers greater ease of use at substantially lower cost while delivering a higher spatiotemporal resolution, with a recent demonstration, in conjunction with contrast agents, of its capability to detect vascular activity with a 6.5-μm spatial resolution (*10*).

Ultrafast ultrasound imaging, however, remains, principally, a two-dimensional (2D) technique due to the stringent spatiotemporal sampling requirements of the imaging process. This imaging process necessitates the transmission of a sequence of planar or diverging waves at high frame rates (≥5 kHz) while recording, in-parallel, the backscattered signals over a surface sampled spatially and temporally at Nyquist rates (*1*). In the case of 3D imaging, this typically requires thousands of elements (as compared with 64 to 256 for 2D imaging) and a corresponding number of independent data channels with associated radio frequency digitizers. Recent works have reported 1024 channel systems for 3/4D cardiac imaging (*11*, *12*), superresolution (*13*, *14*), and functional imaging in rats (*15*). However, these required the use and synchronization of multiple data acquisition systems which are both extremely costly and technically complex, making it infeasible for most clinical applications. To reduce the number of necessary independent data channels, two approaches are conventionally considered: (i) retaining a fully sampled array but using more complex readout schemes using application-specific integrated circuits (ASICs) to combine and prebeamform signals (*16*–*18*) and (ii) sparse arrays that strategically subsample an aperture with an element distribution designed to minimize side lobes (*19*, *20*). However, both methods impose compromises on the image formation process and such arrays are technically complex to realize. Moreover, the small element sizes (on the order of the acoustic wavelength λ ∼ 100 to 300 μm) imposed by Nyquist on all conventional arrays result in poor individual element sensitivity. For hemodynamic imaging, particularly in small animals that suffers from small signal amplitudes, this poses a substantial challenge.

**Fig. 1.**
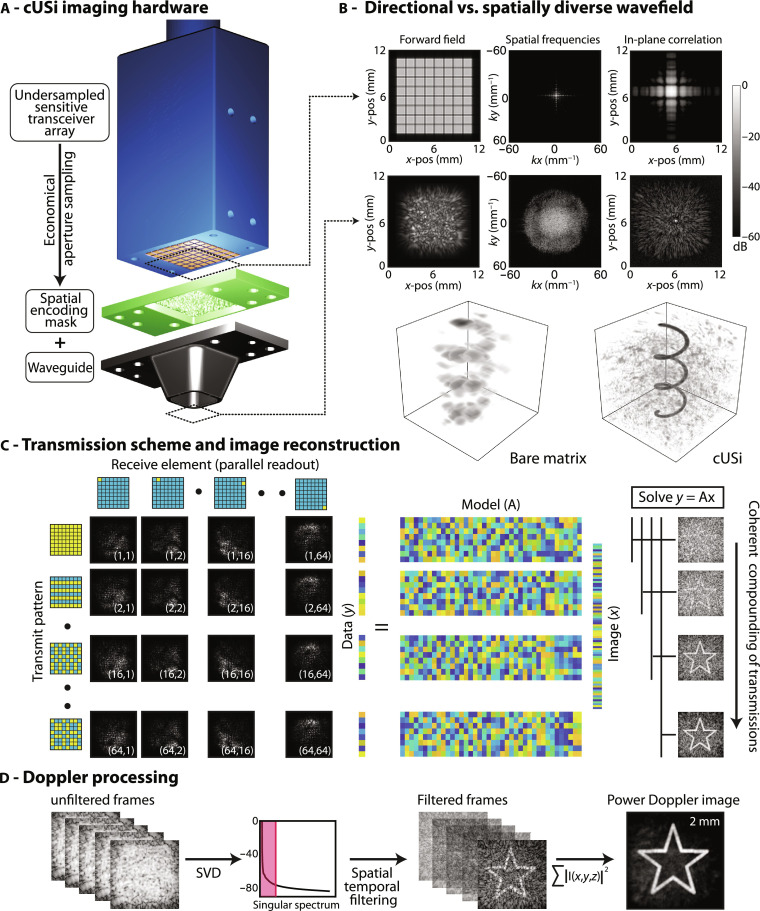
cUSi of blood flow with a matrix probe. (**A**) Rendering of the cUSi imaging hardware. We modified a matrix probe populated with acoustically large, sensitive elements by attaching a plastic encoding mask that scrambles the transmit and receive wavefields. We coupled the mask to a waveguide that confines the transmitted fields and provides the necessary imaging offset. (**B**) Scrambling the transmitted wavefield provides a broader sampling of *k*-space and allows us to trade lateral resolution and side-lobe intensity. We illustrate this with the two renderings, which compare the imaging performance of the bare (without mask) and cUSi (with mask) probe on a numerical spiral phantom occupying an 8 mm × 8 mm × 8 mm volume. We simulated data using the experimentally measured forward fields (i.e., using *y* = Ax) and used a matched filter to reconstruct. (**C**) We drove the probe using a Hadamard-encoded synthetic aperture scheme, signals from different transmissions are separately reconstructed using a model-based approach then coherently summed to form each 3D volume. The system response is calibrated with a one-time measurement and then correlated with each set of measurements to recover an image. (**D**) To generate Doppler images, we continuously transmit to acquire data that are used to reconstruct separate volumes at a rate of ∼400 Hz. The data are spatiotemporally filtered, and the power associated with blood flow in each voxel over time is evaluated to form the PDI.

To address the aforementioned challenges, we introduce computational ultrasound imaging (cUSi). The complex hardware requirements and sensitivity challenges associated with sparse or ASIC addressed arrays are avoided with the use of a simple, fully populated, matrix probe that is undersampled using acoustically large, and therefore highly sensitive, elements. Inherently, these large elements are extremely directional (Fig. 1A) and thus incapable of high-resolution imaging. To compensate for this loss of resolution, we attach a plastic encoding mask and acoustic waveguide to the probe (see Materials and Methods). The encoding mask scrambles the transmitted fields, while the waveguide confines them to our imaging window. By modifying the field in this way, we more evenly sample the *k*-space of our imaging aperture while avoiding any symmetries that would give rise to artifacts such as grating lobes (Fig. 1B) (*21*). This more uniform sampling of *k*-space increases the lateral resolution of the imaging system at the expense of increased clutter/side lobes, which can be seen in the imaging performance of the bare probe versus the probe with mask (cUSi) on a numerical phantom (Fig. 1B). The challenge introduced, however, by imaging with complex wave fields is that it prohibits the use of the conventional, geometry-based, processing (e.g., delay-and-sum) that is routinely used in ultrasound for image formation (*22*). Instead, we reconstruct images using a model-based approach after calibrating the 3D imaging response of our system using a one-time measurement (Fig. 1C and Materials and Methods) (*23*). As in the traditional ultrafast imaging, we coherently compound multiple transmissions to enhance image quality, because each additional transmit-receive acquisition will decrease the spatial correlations between neighboring voxels (fig. S1). Last, we extract information on the 3D flow direction and power using standard Doppler processing techniques (Fig. 1D), e.g., spatial temporal filtering and auto-correlation.

cUSi falls into the category of computational imaging, which covers techniques across a range of modalities that broadly aim to realize cheaper and/or faster imaging devices in part by shifting the burden of image formation from complex hardware onto computation (*24*–*29*). Within the ultrasound domain, the exploitation of reverberant media to reduce the number of sensors required for imaging has been investigated since the 90s (*30*–*33*). However, the translation of these methods to in vivo imaging has, traditionally, been hindered by challenges in separating backscattered signals from the transducer cross-talk as well as the high-sensitivity of these media to small perturbations (e.g., temperature shifts). Recent work has demonstrated the application of reverberant media for 2D in vivo Doppler imaging; however, as with earlier works (*32*), this used separate elements for transmit and receive and required the use of contrast agents due to poor signal-to-noise ratio (SNR) (*34*). In photoacoustics, where backscattered cross-talk is not present, reverberant media have been successfully applied to ultrafast imaging of hemodynamics and functional activity via changes in optical absorption (*35*–*37*). This current work builds on proof-of-principle work on the use of a spatial encoding mask to mitigate sampling constraints (*23*, *38*). Here, we show a practical implementation that translates to 3D in vivo recordings and ultrafast imaging.

## RESULTS

We validated the potential of cUSi for in vivo imaging of brain hemodynamics in both awake and anesthetized mice. In both experiments, imaging was performed through a cranial window, which was covered with a polymethylpentene (TPX) film (CS Hyde Company, IL, USA) in the awake case only. This craniotomy was applied to remove attenuating effects of the skull on the wavefield. In addition, in silica, we found that skull aberrations can have a greater impact when imaging with complex wavefields (note S1 and fig. S2). An 8 by 8–element matrix probe (1.25-mm pitch, 1 cm by 1 cm aperture, 13.8 MHz, Imasonic France) was used for all experiments. This was undersampled by a factor of almost 500 compared to a fully populated array sampled at Nyquist rate of 32,000 sensors. Both the spatial encoding mask and waveguide were fabricated in-house using computer numerical control (CNC). The spatial encoding mask was fabricated from Rexolite 1422 (owing to its favorable acoustic properties), while the acoustic waveguide was fabricated from aluminum to provide a high impedance mismatch with water. The output aperture of the waveguide was 7 × 7 mm to fully confine the transmitted fields to the cranial window. The probe was driven using a synthetic-aperture transmission scheme while applying Hadamard encoding to boost SNR and attain full information from the element array (*39*, *40*). The transmission rate was 32 kHz which, including a short dead time for data transfer and storage, resulted in a volume rate of 407 Hz. We acquired data continuously at this volume rate for 60 s generating a data ensemble from which the Doppler images were formed.

Before image reconstruction, we removed any frames that were subject to jitter or instability. After removal of these unstable frames, the effective volume rate for the awake and anesthetized datasets were 128 and 134 Hz, respectively. We applied a spatiotemporal filter to the stable data ensemble to obtain the signals arising from blood flow, eliminating the large component originating from static soft tissue (Fig. 1D) (*41*). For the anesthetized mouse brain, volumes of size 7.68 mm × 9.6 mm × 8 mm with an isotropic voxel size of 40 μm were reconstructed by correlating the measurements with a calibrated model of the spatiotemporal impulse responses of our imaging system (Materials and Methods) providing a 4D volume ensemble dataset *u*(*x,y,z,t*). To form a power Doppler image (PDI), we computed the average power for each voxel. To evaluate the direction of flow, we computed a lag-1 autocorrelation of the temporal course of each voxel (*42*).

**Fig. 2.**
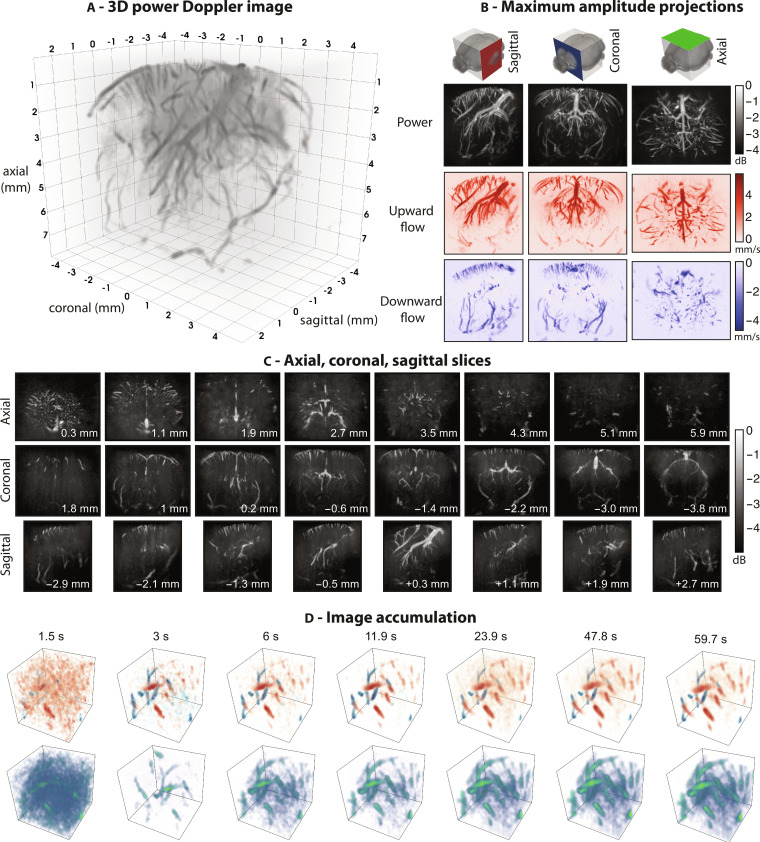
cUSis of hemodynamics in the anesthetized mouse brain. (**A**) 3D rendering of the reconstructed PDI of the anesthetized mouse brain. Image was formed by compounding a filtered dataset composed of 8041 volumes using stable data acquired over 60 s. (**B**) Axial, coronal, and sagittal maximum amplitude projections through the reconstructed power (top) and color (bottom) Doppler volumes. (**C**) Subprojections through the PDI rendered in (A). Each slice was formed from a maximum amplitude projection through a set of planes with a thickness of 480 μm (corresponding to 12 planes in the reconstructed volume). The approximate position of each projection in the brain is denoted on the image. For the coronal slices, the positions are indicated relative to the bregma. (**D**) 3D rendering of vessels (bottom) and flow direction (top) in a fixed 2.48 mm by 2.48 mm by 2.4 mm region of the cortex formed via summation of an increasing number of frames. The time indicated denotes the acquisition time for the number of frames in the stable data ensemble that were used to form the image.

In Fig. 2, we highlight the capability of cUSi for capturing brain hemodynamics in a small rodent model. In Fig. 2A, a 3D rendering of a 6.8 mm by 9.2 mm by 8 mm region of the PDI is shown, where both small and large vessels are resolved throughout the entire cranial window. In Fig. 2B, we feature the information on flow direction that can be extracted from the Doppler ensemble showing draining cortical vessels. Figure 2C shows axial, coronal, and sagittal slices through the power Doppler volume indicating the richness of the small vessels that can be detected, particularly in the cortex, as well as the isotropic lateral resolution that we achieve. Last, Fig. 2D demonstrates the dependency of the imaging performance on increasing Doppler ensemble size. In the current implementation, we require a 3-s acquisition time to form reliable volumes that could be used for other modalities such as fUSi. Lateral, sagittal, and coronal flythroughs of the converged PDI are shown in movies S1 and S2. In fig. S3, the power and color Doppler volumes of an awake mouse are shown. We obtain similar performance, however, there is a loss of fine detail in the cortex and of deeper vessels due to the loss in SNR. This loss of SNR is the result of attenuation generated in the TPX film that we used to cover the imaging window.

## DISCUSSION

Traditionally, the clinical paradigm for ultrasound imaging has focused on robust hardware and simple, fast processing that provides real-time feedback. However, there are clear economical and technical challenges with scaling this approach to 3/4D imaging that limit its use to specific targets (e.g., cardiac imaging). Moreover, there are emerging clinical applications, such as low-cost wearable devices (*43*) and transcranial imaging (*44*), for which this traditional paradigm is less appropriate. In this work, we have demonstrated the possibility for high-resolution 4D imaging of hemodynamics in the mouse brain using cUSi. This represents a proof of concept for such model-based approaches in biomedical ultrasound. While in this work we applied it with a custom-built matrix probe, the approach can be equivalently adapted to existing hardware, for example, row-column arrays.

Despite this successful demonstration of high-resolution imaging of vasculature in the mouse brain, the present cUSi implementation presents several limitations. The principal limitation for this work was the high sidelobes originating from the large factor by which the array was undersampled as well as the bulk attenuation and interfacial losses introduced by the coding mask. The result of both was that we were unable to resolve anatomical features of the brain on the B-mode images, and the dynamic range of the PDIs was low compared to conventional 2D power Doppler. The temporal resolution with which we could resolve the vasculature was similarly limited to approximately 3 s (Fig. 2D) because of these high sidelobes along with the limited volume rates of 134 and 128 Hz in the anesthetized and awake data.

In the future, we could ameliorate these limitations via several modifications. First, the acoustic losses are the result of using a transmissive encoding mask by instead using a rigid (metal) reflector to modulate the matrix field these acoustic losses could be minimized. Alternatively, decoupling of the elements used for transmission and reception [e.g., as in (*34*)] would allow for greater flexibility in material choice. Second, for this proof of concept, we adopted a 64-element probe for imaging; however, we can readily increase this to 256 elements to bring it in-line with contemporary 2D imaging systems. This should allow for a corresponding improvement in imaging quality (movie S3) at the expense of increasing the computational size of the inverse problem. This increased element number would similarly allow for higher temporal resolution by enabling the compounding of fewer transmissions while preserving image quality. The optimal choice for a given application will depend on the imaging/sensing target [e.g., for the measurement of a single physiological parameter very few elements could be used (*43*)]. More broadly, in computational optics, substantial improvements in imaging performance have been achieved via joint optimization of the encoding mask, illumination scheme, and image reconstruction (*45*–*47*). The adaptation of these methods to acoustics rather than the heuristic design approach used in this work provides a route toward low-cost, high-resolution, 3D, structural and fUS devices for different clinical applications.

## MATERIALS AND METHODS

### Animal preparation and surgery

In this study, we assessed cUSi through in vivo imaging experiments in both anesthetized (*n* = 1) and awake (*n* = 1) adult C57BL6/J mice (8 to 10 weeks old). At arrival, animals were group-housed under a 12-hour light/12-hour dark cycle, with controlled temperature and humidity, and with access to water and food ad libitum. After surgery, mice were individually housed. The national authority (Centrale Commissie Dierproeven, The Hague, The Netherlands; license: AVD1010020197846) granted ethical approval before the experiments, which were performed according to the institutional, national, and European Union guidelines.

For both experiments, we performed a craniotomy to facilitate imaging without distorting and attenuating effects of the skull. During surgery and the nonawake imaging experiments, mice were anesthetized using an isoflurane/oxygen mixture (5% induction and 1.752% maintenance), while body temperature was kept constant at 37°C, and heart and respiration rates were monitored (Small Animal Physiological Monitoring System, Harvard Apparatus, MA, USA). The same device was used to fixate and level the head while drilling (Foredom) the cranial window. For the anesthetized imaging experiments, a cranial window of 1 cm by 1 cm was made. For the awake experiments, a smaller cranial window (+2 mm by −4 mm from bregma and ±4 mm in width) was performed due to the placement of a pedestal (1 cm by 0.8 cm), which ensured head fixation in the experimental setup during imaging. In the awake case only, the cranial window was covered with a TPX film (CS Hyde Company, IL, USA), and postoperative mice received 3 to 5 days of antibiotics (Baytril, 25 mg/ml; Bayer, Germany) to prevent inflammation of the brain. Before the start of the imaging experiments, both the exposed and covered brain tissues were sprinkled with saline solution, after which ultrasound transmission gel (Aquasonic 100, Parker Laboratories, NJ, USA) was applied for acoustic contact between the brain and the 8 by 8–element matrix probe positioned straight above the cranial window.

### Matrix probe

Experiments used a custom built 64-element matrix probe (fig. S4) [8 × 8 square elements, 1.25-mm lateral width, 10 mm by 10 mm aperture, 13.8-MHz central frequency, 65% −6-dB bandwidth (BW); Imasonic, France]. The element size was approximately 132λ^2^, substantially larger than both the 16λ^2^ used for 1D linear arrays (which while sampled close to Nyquist rate in-plane have a large elevational size) and the 4λ^2^ used previously for 3D functional imaging of rats (*15*). The front surface of the probe had a 15 mm by 25 mm footprint and included tapped holes on each corner for fixation of coding masks to the front surface.

### Spatial encoding mask

For cUSi, we aim to perturb the field for each matrix element by introducing a spatially varying phase shift analogous to an (optical) spatial light modulator or acoustic hologram (*48*). We achieve this using a plastic coding mask that introduces a local, thickness-dependent delay to the transmitted field.

The coding mask material, ideally, would fit several criteria. Namely, impedance matching to water and low acoustic attenuation to minimize insertion losses, high-sound speed contrast to soft tissue to maximize phase delays, and mechanically machineable/castable/printable with high precision. Rexolite 1422 was selected to balance these competing requirements as it has low acoustic attenuation and good acoustic matching to water (soft tissue) while being sufficiently rigid to machine (*49*).

The mask had a thin, smoothly varying profile inspired by optical diffusers previously applied for 3D optical imaging (*24*). We generated a smoothly varying surface profile with an average lateral feature size of approximately 370 μm and a total height variation of 1.2 mm. The lateral feature size was constrained by the manufacturing method and the tool size, while the ideal height scaling was determined empirically. We found that for masks that were too thin, we insufficiently modulated the field of each element resulting in a broad point spread function (PSF) that was unable to resolve smaller vasculature. For masks that were too thick, the increased losses due to greater internal reflection and increased absorption resulted in an SNR that was too low for imaging.

The encoding mask design challenge is like that of sparse array design in ultrasound where considerable effort is often devoted to optimizing the element configuration before fabrication (*20*). With cUSi, however, we can retrospectively (and cheaply) modify our transmit/receive fields and therefore the side-lobe distribution, simply by redesigning the encoding mask. While in this work we empirically designed our encoding mask, in the future, inverse-design principles (*45*) could be adopted to refine this approach by incorporating the system physics and reconstruction nonlinearities into the encoding mask optimization (*46**,* *47*).

The encoding mask was generated for a 12 mm by 12 mm area including a 1-mm lateral buffer around the active surface to eliminate sharp discontinuities that would create diffraction effects. This profile was exported as a point cloud to SolidWorks (SolidWorks 2018, Dassault Systemes, V’elizy-Villacoublay, France) where it was converted to a solid part that was interpretable to the CNC software. A 0.4-mm axial buffer was added to the mask profile for mechanical stability, and its aperture was expanded to match the waveguide opening. To attach the mask to the probe, a 2-mm thick, 15 mm by 35 mm buffer was added around the encoding mask including four clearance holes to fixate it to the probe. The mask was then fabricated using a CNC machine (P60 HSC, Fehlmann) using a 200-μm drill bit. A photograph of the coding mask can be seen in fig. S4.

### Waveguide

We added a tapered waveguide to the coding mask to further modify the transmitted wavefield. We introduced this for two reasons. First, the waveguide allows for a controlled offset between the probe surface and the imaging medium. From analysis of the system matrix, the PSF was found to degrade substantially close to (<6 mm) the probe (fig. S5A). This occurs as the field of each matrix element needs to propagate a certain distance after the encoding mask to diverge such that it overlaps with that of neighboring elements. Close to the probe, each voxel is seen by as few as four elements, which results in an extremely poorly conditioned reconstruction. To eliminate this, an offset between the probe and imaging target is required with a distance determined by the divergence to the field introduced by the encoding mask and the element size. For this work, we found 12 mm to be sufficient. Second, the cranial window that can be safely introduced to the mouse’s skull is limited, sagitally and laterally, to ∼8 mm by 6 mm, which is smaller than the probe aperture (10 mm by 10 mm). As such, a large fraction of the transmitted energy (≥40%) falls outside the imaging window. This is compounded by the additional divergence introduced by the encoding mask. With the addition of a waveguide, this energy can be funneled onto the desired imaging window (fig. S5B).

The waveguide had input and output apertures of 14 mm by 14 mm and 7 mm by 7 mm, respectively, and a length of 10 mm. The minimum length was set by the discussed need for an offset for the imaging target as well as the requirement to avoid a sharp taper angle that would trap waves within the waveguide and reflect them toward the probe surface. There is no physical constraint on maximum length; however, an overly long waveguide suffers greater losses from absorption, and practically, is more prone to trapped air pockets in the ultrasound gel, which modify the system response from the calibration. We fabricated the waveguide from aluminum using CNC micro-machining. Aluminum has a much higher acoustic impedance than both water and ultrasound gel (18 versus 1.5 MRayls) so near fully confines the transmitted field (fig. S5B).

### Transmission scheme

We adopted a synthetic aperture transmission scheme (*39*) [also referred to as full-matrix capture; (*50*)] for imaging. Each element sequentially transmits while recording the backscattered signals on all the array elements in parallel, allowing for each voxel in the imaging medium to be synthetically focused on during reconstruction. For the probe in this work, each volume is therefore formed with a set of 64 transmissions. The principal disadvantage of synthetic aperture is poor SNR as each transmission only uses a single element. To improve this, we applied spatial coding on transmit (*40*). For each of the 64 transmissions, a different spatial code was applied to the matrix probe elements with each code forming a column of a 64 × 64 invertible matrix. We chose a Hadamard matrix as it is composed of ±1s so is easily implemented in hardware by flipping the polarity of the driving signal. By transmitting on all 64 elements each time, we gain a factor of $\sqrt{64}$ or ×8 improvement in SNR. For conventional ultrasound, it is necessary to decode the signals before reconstruction by applying the inverse of the Hadamard matrix. However, our image reconstruction fully models the relevant wave physics, so this step is not required. Instead, we incorporate the spatial codes into our model of the transmission.

It would be equally feasible to use cUSi with alternative transmission schemes. For example, sequential focusing on each point in the imaging medium via time reversal (*33*). While this would result in greater SNR, it would not be compatible with the volume rate necessary for ultrafast imaging of blood flow. Alternatively, synthesis of nondiffracting planar or diverging waves behind the mask would similarly allow for a trade-off of frame rate versus contrast while simplifying the image reconstruction; however, the transmit side lobes are too high to realize these with our system. In future work, direct optimization of the transmit scheme given knowledge of the system matrix would similarly provide the best trade-off between frame rate and image quality for a given application.

### Experimental setup

The probe, encoding mask and waveguide were each submerged and then assembled underwater using the four tapped holes on the front surface of the probe (Fig. 1A). Before assembly, if small air pockets were present on the mask, they were manually removed from the encoding mask surface under a microscope using a syringe. Ultrasound coupling gel was then syringed into the end of the waveguide while still submerged to allow it to retain water when vertically mounted. Before application, this syringe containing gel was centrifuged at 6000 rpm for 5 min to remove bubbles (*51*). The probe was then suspended vertically above the cranial window and lowered onto the exposed mouse brain using a manual translation stage. Before imaging, we assessed whether any air pockets were present in the waveguide by transmitting an impulse from each element and recording the backscattered signals. The backscattered signals were analyzed to confirm that no signals originated within 10 mm of the probe surface.

### Experimental measurements

The probe was driven using an ultrasound research system (Vantage 64LE, Verasonics Redmond, WA, USA) with a 5-cycle toneburst centered on 15.625 MHz. A single transmit sequence was used for both the awake and anesthetized mouse imaging experiments. The pulse repetition frequency was 32 kHz, giving an underlying volume rate of 500 Hz. Transmissions were repeated in blocks of 6400 firings corresponding to 100 volumes. Between each block, a dead time of approximately 50 ms was used to transfer the raw data buffer and write to a hard disk. The scheme was repeated for 60 s for both experiments resulting in 24400 volumes and an effective volume rate of 407 Hz.

### Data preprocessing

Signals were sampled in 50% BW mode to reduce data requirements. For each transmission, 512 samples at 50% BW (*52*) corresponding to an imaging depth of 24.6 mm were recorded on each element. The 50% BW data were then Fourier-transformed and downsampled by a factor of 2 to generate a set of 128 frequencies *N*_ω_ that were used for reconstruction. These were evenly spaced between 11.7 and 19.4 MHz (i.e., a 50% BW centered on 15.625 MHz).

Next, any frames subject to jitter from breathing or other motion were removed by evaluating a lag-1 difference over sequential frames and filtering the frames falling above an empirically determined threshold. At this stage, an instability resulting from hardware was identified that necessitated the removal of the first 64 frames from each block of 100 from both datasets. For the awake and anesthetized measurements, respectively, this left a total number of frames *N_f_* of 8041 and 7679 frames.

We then used a clutter filter to separate the static or quasi-static signals corresponding to tissue from those originating from blood, which was accomplished using a singular value decomposition (SVD) (*41*). First, both acquisitions were reshaped into a 2D matrix with dimensions (*N*_ω_ × *N_t_* × *N_e_*, *N_f_*), where *N_t_* is the number of transmissions and *N_e_* is the number of elements. Next, the SVD was evaluated over this full set of frames and the data above a manually determined threshold cutoff. For both datasets, this threshold was set at 65% of the singular values (e.g., for the anaesthetized dataset, the first 5226 values were discarded). It should be noted that this spatiotemporal filtering is typically applied after image reconstruction; however, we found no major difference in applying it to our data as a preprocessing step. After this preprocessing, our data consist of a 4D tensor **v** with dimensions (*N*_ω_, *N_t_*, *N_e_*, *N_f_*).

### Acoustic model

Conventional ultrasound images are reconstructed geometrically. Simple, nondiffracting, spherical or planar fields are transmitted allowing for images to be reconstructed using only knowledge of the sensor positions and the speed of sound in the propagation medium. The use of a coding mask and waveguide prohibits this with cUSi as the wavefield evolves in a complex manner with propagation. Instead, we assume that our data **v** can be linearly related to a 3D image **u**, via a matrix-vector multiplication

v=Au (1)

Here, we assume that the tensors **u** and **v** are vectorized, and the matrix **A** contains the pulse-echo impulse response to our transmission scheme for each voxel in the imaging medium. The matrix **A** has number of rows equal to *N*_ω_*N_t_N_e_* and number of columns equal to the number of voxels in the reconstructed image. Reconstructing an image requires precise knowledge of this matrix **A**. In addition, as the dimensions for **A** in this work are 8 by 10^6^ by 5 by 10^5^, it occupies over 30 Tb in memory so cannot be stored. Hence, we construct and apply it sequentially.

To construct **A**, first we perform a one-time experimental calibration of the forward field of each element. The probe with the encoding mask and waveguide attached was mounted in a custom-built scanning tank with a three-axis computer-controlled positioning system formed from three translation stages X-LSM200B, XLSM100B, and X-LDA075A (Zaber, Vancouver, Canada) and driven using the ultrasound research system. The impulse response for each element was measured over a 12 mm by 12 mm plane parallel to the end of the waveguide with a 40-μm spacing using a broadband 0.2-mm needle hydrophone (Precision Acoustics, Dorchester). Signals were sampled using a programmable analog-to-digital converter (M4i.4450-x8, Spectrum, Germany) with 14 bits per sample and a 250-MHz sampling rate. The forward field for each of the 64 elements was measured in a single scan. To improve SNR, signals were averaged eight times at each position, to reduce the amount of averaging required the probe was driven using the Hadamard encoded synthetic aperture scheme used for imaging. To measure the probe’s full BW, it was driven using a 32-Vpp 32-ns impulse; however, to further reduce the required averaging, we used temporal Golay codes following Bae *et al.* (*53*). After measurement, both the temporal and spatial encoding were deconvolved. The calibration took approximately 6 hours and generated a set of 64 spatial temporal measurements *p_N_e__*(*x*, *y*, *z_s_*, *t*), where *z_s_* is the plane over which the calibration was performed. These wavefields were then mapped onto the 128 frequencies *N*_ω_ that comprised the experimental data. The same calibration was used to reconstruct both datasets, acquired on separate days, demonstrating that the calibration is robust to repetition. Maximum amplitude projections through each of the elements forward fields are included as movie S4.

The use of a 200-μm hydrophone for the calibration, which is larger than the acoustic wavelength, results in an underestimation of the higher spatial frequencies of the forward field due to its directivity. This hydrophone was chosen after comparison with a calibration performed using a 40-μm needle hydrophone using the same number of averages. The reconstruction of the anesthetized dataset using the calibration performed using the 40-μm hydrophone compared with the 200-μm needle hydrophone can be seen in fig. S6. The 40-μm reconstruction has lower contrast due to the increased noise in the calibration measurement, which is the result of its lower sensitivity from its smaller area. This lower sensitivity could be compensated for by using more averaging; however, this results in a corresponding increase in scan time, risking changes in the scan conditions. As we found no substantial structural differences between the images generated with the two calibration measurements using our reconstruction approach, the 200-μm calibration was used. In the future, the directivity and other errors associated with the experimental calibration of **A** used here could be eliminated with the use of blind calibration methods (*54*) or by deconvolution of the directivity response (*55*).

We assume that the propagation is linear, allowing us to predict the forward field at a location (*x*, *y*, *z_s_*) for a frequency *n*_ω_ for any arbitrary spatial apodization vector **H**_**N_e_**_ and temporal delay vector **T**_**N_e_**_ applied on transmission as a simple weighted summation

$$p\left(x,y,z_{s},n_{\omega }\right)=\sum_{n_{e}=1}^{N_{e}}H_{n_{e}}p_{n_{e}}\left(x,y,z_{s},n_{\omega }\right)e^{-i2\pi n_{\omega }T_{n_{e}}}$$ (2)

In our case, we apply no delays to any of the elements and simply use a 64 × 64 apodization matrix for our transmissions $H_{N_{e}}^{N_{t}}$. So, the forward field for any of the transmissions *n_t_* can be calculated simply as

$$p_{n_{t}}\left(x,y,z_{s},n_{\omega }\right)=\sum_{n_{e}=1}^{N_{e}}\;H_{n_{e}}^{n_{t}}p_{n_{e}}\left(x,y,z_{s},n_{\omega }\right)$$ (3)

In addition, reciprocity means that we can assume that each element in the matrix probe behaves identically on transmit as on receive. Therefore, the pulse-echo impulse response for an element *n_e_* for a transmission *n_t_* for a position in the calibration plane can be calculated via temporal convolution reducing to a multiplication in the frequency domain.

This allows us to evaluate **u** = **A**^H^**v** for any voxel in the calibration plane *n_s_* for a frame *n_f_* via

$$u\left(x,y,z_{s},n_{f}\right)=\sum_{n_{\omega }=1}^{N_{\omega }}\sum_{n_{e}=1}^{N_{e}}\sum_{n_{t}=1}^{N_{t}}\left\{p_{n_{e}}\left(x,y,z_{s},n_{\omega }\right)p_{n_{t}}\left(x,y,z_{s},n_{\omega }\right)\right\}\times v\left(n_{\omega },n_{t},n_{e},n_{f}\right)$$ (4)

and, similarly, **v** = **Au** can be evaluated as

$$v\left(n_{\omega },n_{t},n_{e},n_{f}\right)=\int \int \left\{p_{n_{e}}\left(x,y,z_{s},n_{\omega }\right)p_{n_{t}}\left(x,y,z_{s},n_{\omega }\right)\right\}u\left(x,y,z_{s},n_{f}\right)\text{dxdy}$$ (5)

To reconstruct other depths, we use the angular spectrum method to project the individual forward fields. For an acoustic field over a 2D plane *p*(*x*, *y*, *z_s_*) at a frequency *f*, this calculates the field over a parallel plane at a depth *z_d_* = *z_s_* + *d* as

$$p\left(x,y,z_{d}\right)=F_{k_{x}k_{y}}^{-1}\left\{F_{\mathrm{xy}}\left\{p\left(x,y,z_{s}\right)\right\}∙H\left(k_{x},k_{y},d\right)\right\}$$ (6)

Here, $F_{xy}$ and $F_{k_{x}k_{y}}^{-1}$ denote the 2D discrete Fourier and inverse Fourier transforms, respectively, and *H*(*k_x_*, *k_y_*, *d*) is a propagator function given by

$$H\left(k_{x},k_{y},d\right)=\frac{e^{-\mathrm{id}\sqrt{k_{x}^{2}+k_{y}^{2}-k^{2}}}}{i\sqrt{k_{x}^{2}+k_{y}^{2}-k^{2}}}$$ (7)

where *k* is the wave number. Following Zeng and McGough (*56*), we apply an angular cutoff to the propagator

kc=kD22D22+d2 (8)

to eliminate the undersampled and evanescent spatial frequencies, where *D* is the lateral dimension of the propagated field.

Practically, for reconstruction, it is necessary to discretize our imaging domain. For the images in this work, a 40 μm by 40 μm by 40 μm spacing was used matching the calibration measurement (to avoid resampling) and corresponding to a spacing of $\frac{\lambda }{2.4}$. The image domain for the images in Fig. 2 was 7.68 mm by 9.6 mm by 8 mm, while for the images in fig. S3, it was 7.04 mm by 6.24 mm by 7.2 mm. Last, calculation of **u** = **A**^H^**v** and **v** = **Au** proceeds by first calculating the field for each transmission over the current depth using Eq. 3. Next, **A**^H^**v** or **Au** is evaluated for the current depth using Eq. 4 or 5. Last, for each of the individual elements, forward fields are stepped to the next depth using Eq. 6, and the process is repeated for this depth.

### Image reconstruction

Our image reconstruction was formalized in a set of linear equations, **v** = **Au**, allowing a variety of different solvers to be used for reconstruction. We compared three different methods.

The first method was the least squares estimate, which finds an image $\hat{u}$ minimizing the square error between the modeled **Au** and the measured data **v** as follows

$$\hat{u}=\text{argmi}n_{u\prime }{\left\|v-\text{Au}\prime \right\|}_{2}^{2}$$ (9)

This was implemented using the LSMR algorithm (*57*), similar to the LSQR algorithm (*58*), which is regularly used for large-scale sparse systems, however, safer to use in the case of early termination that was necessary here. Coronal maximum amplitude projections through the complete PDI and a fixed 800-μm slice of the volume for increasing LSMR iteration are shown in fig. S7. The underlying vasculature can be clearly resolved in each image; however, for early iterations, there are large variations in the vessel intensity and background caused by spatial variation in the sensitivity of the underlying model (*59*). These are compensated for by later iterations, and beyond iteration 8, there is minimal change in the reconstructed volume aside from a gradual loss of contrast as the solver fits the noise in the data and errors in the underlying model. This has been reported previously (*23*), however, occurs more rapidly for the in vivo data reconstructed here. We attribute this more severe noise amplification to the lower SNR present in ultrafast imaging compared to the sparse, rigid, objects imaged in previous work.

To try to alleviate the loss of contrast, we also tested a sparsity promoting reconstruction method, choosing the iterative two-step shrinkage/thresholding algorithm (TwIST) (*60*), which minimizes the following cost function

$$\hat{u}={\text{argmin}}_{u\prime }{\left\|v-\text{Au}\prime \right\|}_{2}^{2}+\lambda {\left\|u\prime \right\|}_{1}$$ (10)

Here, λ is a dimensionless scalar weighting the regularization, and ‖**u**′‖_1_ is the l1-norm. The regularization parameter was chosen empirically as λ = 0.2 max (**A**^**H**^**v**). After convergence of the initial algorithm, we applied a debiasing step to the nonzero elements of **u** using a conjugate gradient method following Figueiredo *et al.* (*61*). However, TwIST with debiasing was found to be effectively equivalent with the early iterations of LSMR (fig. S7B), which we attribute to the lack of spatial sparsity in the individual frames used to form each PDI.

Both algorithms share two drawbacks. First, the calibration of the model **A** was performed using a 200-μm needle hydrophone, which, as stated earlier, underestimates the higher spatial frequencies in the forward fields due to being larger than the acoustic wavelength. This results in a model that is less divergent than the actual experimental field and biases against vessels in the periphery of the volume. Second, given the large size of the model matrix **A**, iterative approaches that require it to be repeatedly constructed and applied are time consuming to evaluate. These drawbacks motivated the use of a third approach, which we applied for the results presented in both Fig. 2 and fig. S3. Taking inspiration from observations on one-bit time reversal where the amplitude information was superfluous (*31*), we modified **A** to contain only the phase information [i.e., **A**_new_ = *e*^arg(**A**)^] for each voxel and reconstructed images using a matched filter, i.e., $u=A_{\text{new}}^{H}v$. A comparison of this method with TwIST and LSMR is shown in fig. S7B. Taking only the phase of the model reweights the sensitivity to unity for each voxel. This reweighting is able to partly compensate for the directivity errors introduced by the measurement method at the expense of diminished contrast. In addition, this approach is substantially faster requiring only a single evaluation of **A**^H^**v**.

Once the full set of frames *N_f_* were reconstructed for both datasets, we formed the PDIs from a simple summation of the power in each individual voxel as $\text{PDI}\left(x,y,z\right)=\sum_{n_{f}=1}^{N}$|*u*(*x*, *y*, *z*, *n^f^*)|. The color Doppler images (CDIs) shown in Fig. 2B and fig. S3B were calculated as $\text{arg}\left[\sum_{n_{f}=1}^{N}u\left(x,y,z,n_{f}\right)u\left(x,y,z,n_{f}+1\right)*\right]$. However, we found that the CDI were noisy when we applied this to volumes reconstructed from separate groups of 64 transmissions. Instead, we reconstructed a separate subframe for each set of eight transmissions. A running average was then evaluated over these subframes to create a stack of 8 *N_f_* images each formed from 64 orthogonal transmissions with a lag of 8 transmissions between images. The CDI processing was then applied to this new, larger image stack.

Here, we have shown that with a simple, quick, reconstruction method, we are still able to form high-resolution 3D images of the vasculature. In the future, there is scope to apply more advanced reconstruction methods that exploit statistical independence of the signals in neighboring voxels (*62**,* *63*).

### Computational complexity

The use of a model-based inversion scheme increases the computational complexity of the image reconstruction in comparison with conventional delay-and-sum–based beamforming. The stored data for both the anesthetized and awake datasets occupied 156 GB in memory. After filtering of unstable frames and transformation to the frequency domain, this was reduced to 33.7 and 32.2 GB, respectively. The planar forward fields for each of the 64 elements from the calibration scan used for the slice-by-slice construction of the model matrix **A** occupied 4.1 GB, while the overall size of the acoustic model **A** that was computed with this approach was 40 TB. The power and color Doppler volumes presented in Fig. 2 and fig. S3 were formed directly slice by slice, avoiding storage of the intermediate beamformed volumes to reduce memory requirements. Reconstructions were performed on a computing cluster with 2 Intel Xeon Silver 4314 2.4 GHz 16 core processors, 1024 GB of 2666 MHz memory, and 3 Nvidia A100 80 GB graphics processing units (GPUs). A single GPU was used for all reconstructions. The reconstruction was implemented in MATLAB software, release 2021a (The MathWorks). Reconstruction of 8,041,192 × 250 × 200 volumes took approximately 7.7 hours on this hardware.

### Image resolution

We approximated our image resolution in three distinct ways. First, we calculated the decay rate of the lateral and axial correlations of our system matrix **A** at three different depths. Second, we approximated the length scale on which we can distinguish separate cortical vessels over a fixed depth. Last, we analyzed the lateral and axial correlations of the system matrix over planar cross sections covering the full field of view. These are illustrated in fig. S8.

The correlations were evaluated at depths corresponding to 1, 4, and 7 mm inside the mouse brain in the experimental data. In each case, we selected a single voxel, and the magnitude of the correlation coefficient of this voxel with each voxel of the system matrix **A** over a 2 mm–by–2 mm–by–1 mm region with a 40-μm spacing was calculated. The results demonstrate that both the lateral and axial resolution decrease with imaging depth; however, this occurs more slowly for the axial resolution. The axial resolution is higher than the lateral resolution, as is commonly the case for ultrasound imaging. Higher side lobes are seen in the axial direction; however, these are largely confined to a narrow line in the depth direction. By zooming in on a fixed axial slice of the reconstructed PDI, we can see that this correlation-based approximation of the lateral resolution corresponds well with the scale of structures reconstructed from the experimental data. Vessels in the cortex are resolvable through the Rayleigh criterion (*24*) with a spacing of less than 200 μm. Last, calculating the correlations over an axial and three lateral cross-sectional fields of view on a 200-μm grid spacing in the mouse brain confirms the local volumetric estimates. In depth, the axial resolution varies slowly (fig. S8C), falling from 97 to 105 μm on average over 8 mm. The lateral resolution shows more variation in depth—falling from an average of 115 to 174 μm from 1 to 7 mm (fig. S8, D to F). The lateral resolution also shows some variation over a fixed depth with a large increase occurring toward edge of the field of view on each depth. We also calculated the peak secondary side lobe for each position (fig. S8, G to J). These follow similar trends in plane to the resolution, however, less variation laterally between depths (the peak side lobes for 1, 4, and 7 mm are −20.9, −19.5, and −21.1 dB), while the axial side lobes see an increase (from an average of −18.3 to −16 dB). This axial trend can also be seen in the volumetric cross sections (fig. S8A).

## Supplementary Materials

### This PDF file includes:

Text S1 Figs. S1 to S8 Legends for movies S1 to S4 References
