# Supplementary material for: Four-dimensional computational ultrasound imaging of brain hemodynamics
Source: Sci Adv. 2024 Jan 17;10(3):eadk7957. doi: 10.1126/sciadv.adk7957 (PMC10793943; doi:10.1126/sciadv.adk7957)
Supplement: Supplementary file 1 — Text S1 Figs. S1 to S8 Legends for movies S1 to S4 References [file sciadv.adk7957_sm.pdf]

Science Advances
AAAS

Supplementary Materials for

Four-dimensional computational ultrasound imaging of brain hemodynamics

Michael D. Brown *et al.*

Corresponding author: Michael D. Brown, m.brown@erasmusmc.nl

Sci. Adv. **10**, eadk7957 (2024)
DOI: 10.1126/sciadv.adk7957

The PDF file includes:

Text S1
Figs. S1 to S8
Legends for movies S1 to S4
References

Other Supplementary Material for this manuscript includes the following:

Movies S1 to S4

Text S1. Investigating the effect of acoustic aberrations on cUSi

For this work we conducted imaging through a cranial window that was either uncovered or covered by a thin TPX film. This was done to reduce the attenuation. Separately we performed a numerical experiment to assess whether cUSi is impacted to a greater degree by errors in acoustic properties than traditional imaging. For the numerical experiment we simulated imaging using a 1D linear array through a representative model of a mouse skull: average thickness 450 um, sound speed and density 2250 ms^{-1} and 1950 kgm^{-3} following Estrada et al *(64)*. The linear array was comprised of 60 elements: centre frequency 15.625 MHz, pitch 100 μm. Two cases were simulated: one in which the array was uncovered, and one in which it was covered by a random mask with thickness 1.2 mm and lateral feature size of 340 um to match the experimental mask. Simulations were carried out on a 6x8 mm grid with a spacing of 25 um using the k-Wave toolbox *(65)*. Three sets of simulations were conducted. First, the forward field for each element in free space was simulated to construct the model matrix **A.** Second, we simulated a synthetic aperture pulse-echo imaging sequence in free space. Finally, we simulated a synthetic aperture pulse-echo imaging sequence through the mouse-skull aberrator. For both imaging experiments the target phantom was a regular grid of points. Images were reconstructed by applying both a matched filter and LSQR using the free-space model matrix. The resulting reconstructed image can be seen in Fig S2.

The image generated using ‘random’ wave-fields is more noticeably impacted by the presence of the skull compared to the bare linear array resulting in a greater distortion of the reconstructed points, particularly in the near-field, as well as an increase in side-lobes. The grid of points can still be seen in both cases. When applying LSQR, however, using the erroneous model, even in the absence of noise we observe significant artefacts as it rapidly starts to fit errors in the model matrix. This occurs both with and without the coding mask. These results suggest that imaging using cUSi through an intact skull with the matched filter approach employed in this work should be possible, however, for other reconstruction methods some method of correcting the system matrix would be required. For example, thin-phase screen models *(66)* or blind-calibration approaches *(67)*.

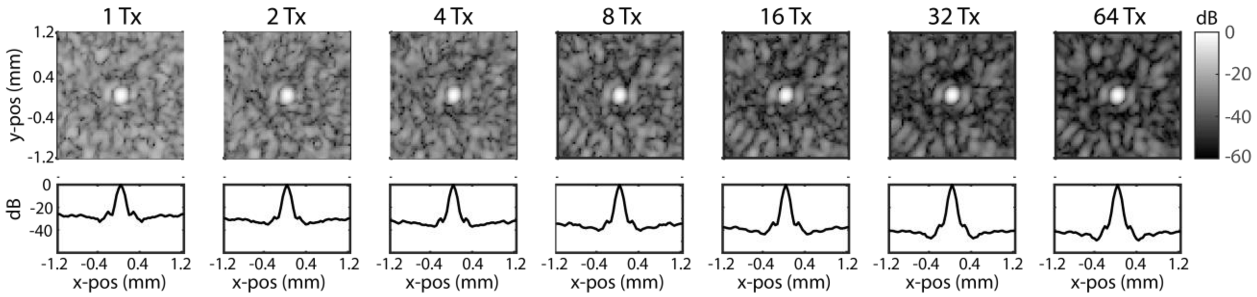

Fig S1. Correlations in system matrix reduced by coherent compounding of transmissions.
Lateral correlations for a voxel at a fixed (x,y,z) position for increasing transmission number.

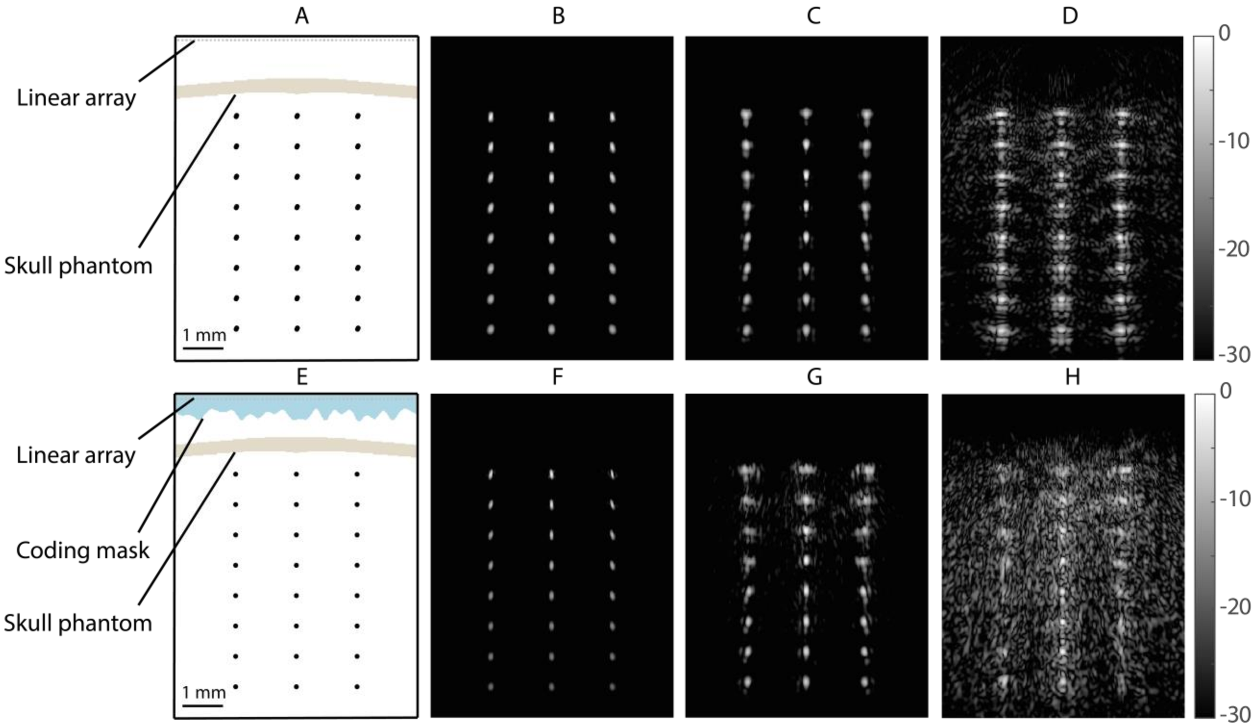

Fig S2. Acoustic aberrators have a more distorting effect on cUSi.

Investigation of the effect of an intact skull on cUSi vs traditional imaging. (**A**) Setup of simulation domain for traditional imaging. (**B-D**) Reconstruction of a numerical phantom comprised of a grid of points in 3 cases: (B) where no aberrator is present and the model is perfect reconstructing using matched filter. (C) Where an aberrator is present using the free-space model and matched filter, (D) where an aberrator is present using LSQR. (**E**) Setup of the simulation domain for cUSi. (**F-G**) Reconstruction of a numerical phantom comprised of a grid of points in 3 cases: (F) where no aberrator is present, the model is perfect, and a mask is attached reconstructing using matched filter. (G) Where an aberrator and a mask is present using the free-space model and matched filter, (H) where an aberrator and a mask is present using LSQR.

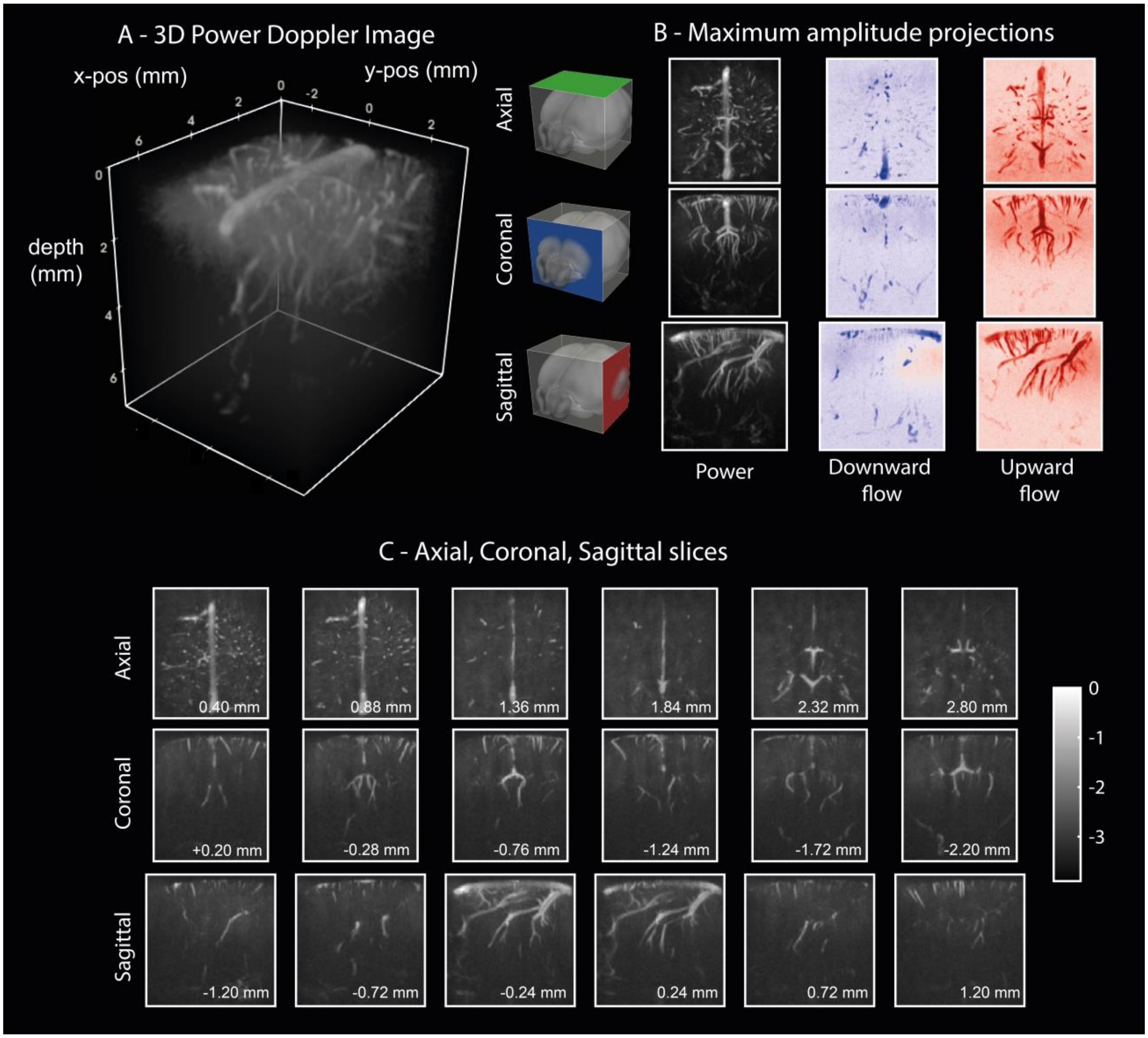

Fig. S3. Computational ultrasound images of hemodynamics in the awake mouse brain.

Results of hemodynamic imaging in the awake mouse brain. **(A)** 3D rendering of the reconstructed PDI of the awake mouse brain. Image was formed by compounding a filtered dataset comprised of 7679 volumes. **(B)** Axial, coronal, and sagittal maximum amplitude projections through the reconstructed Power (left) and Colour (right) Doppler volumes. **(C)** Sub- projections through the PDI rendered in (A). Each slice was formed from a maximum amplitude projection through a set of planes with a thickness of 480 μm (corresponding to 12 planes in the reconstructed volume). For the coronal slices the positions are indicated relative to Bregma. Compared to the anesthetized data shown in Fig. 2 the awake data resolves a lower number of cortical vessels and less vessels deeper in the brain due to the attenuation generated by the TPX film. The field of view is also smaller due to the smaller cranio window.

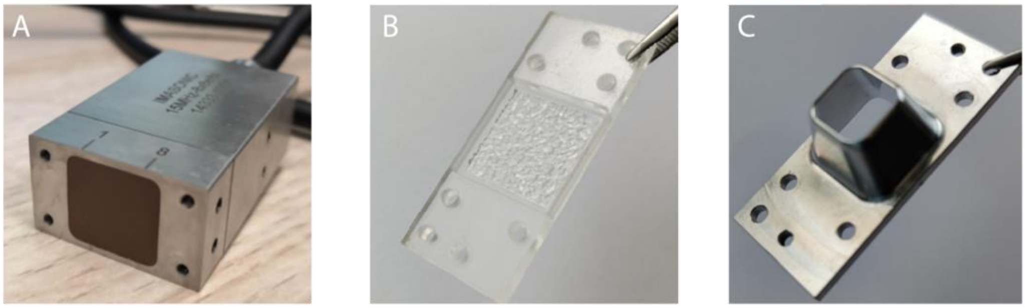

Fig. S4. Photographs of cUSi system components.

(A) photo of the 8×8 matrix probe used for the imaging experiments. (**B)** photo of the Rexolite spatial encoding mask fabricated using CNC micromachining. (**C**) photograph of the Aluminum waveguide fabricated using CNC micromachining.

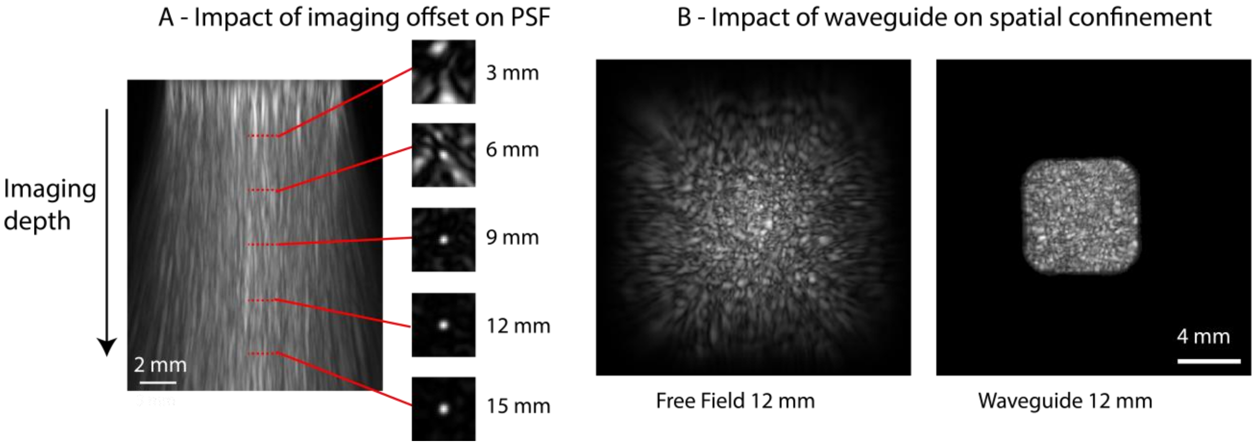

Fig. S5. Acoustic waveguide provides a required imaging offset and confines wavefield.
(A) Impact of imaging off-set on the point spread function. The top image is a maximum amplitude projection of a field at 15.6 MHz experimentally measured through an example coding mask while transmitting a plane wave. For this measurement no wave-guide was added to the coding mask so the field freely diverges. The 5 images on the bottom show the (normalised) correlations for a fixed (x,y) pixel at 5 different depths (3, 6, 9, 12, 15 mm) from the front surface of the probe. These correlations were analysed for a synthetic aperture transmission scheme as employed in the experimental measurements. For planes close to the imaging probe the lack of overlap between the fields of neighbouring elements makes it impossible to resolve echoes from distinct (x,y) locations. **(B)** Experimentally measured fields for a coding mask without (left) and with (right) a waveguide transmitting a plane-wave on the matrix probe. The waveguide effectively confines the transmitted field to the desired imaging aperture.

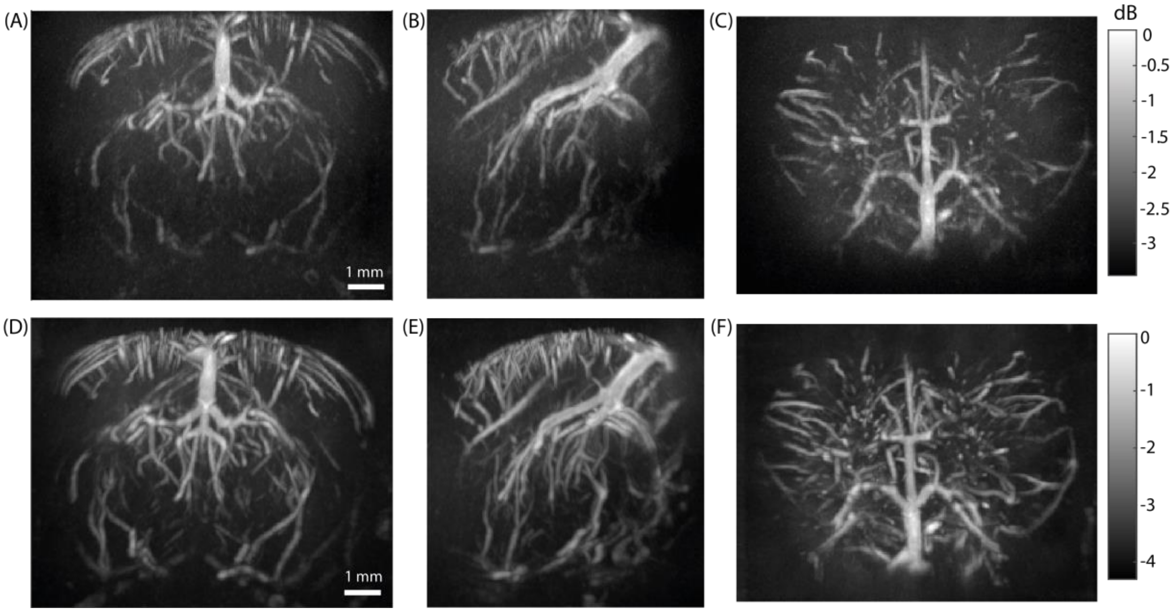

Fig. S6. Comparison of imaging performance using calibrations from a 40 vs 200 µm needle hydrophone.

(**A-C)** Maximum amplitude projections through PDIs reconstructed using calibration of the cUSI probes forward field using a 40 um needle hydrophone. (**D-F**) Maximum amplitude projections through PDIs reconstructed using calibration of the cUSI probes forward field using a 200 µm needle hydrophone. We performed both reconstructions on the same set of filtered data that was used to form the projections shown in Fig. 2B. The higher noise level in the 40 µm calibration results in correspondingly higher noise in the reconstructed volume. Identical structural features are seen in both demonstrating that there is limited utility in using a smaller hydrophone with the reconstruction approach used for this work.

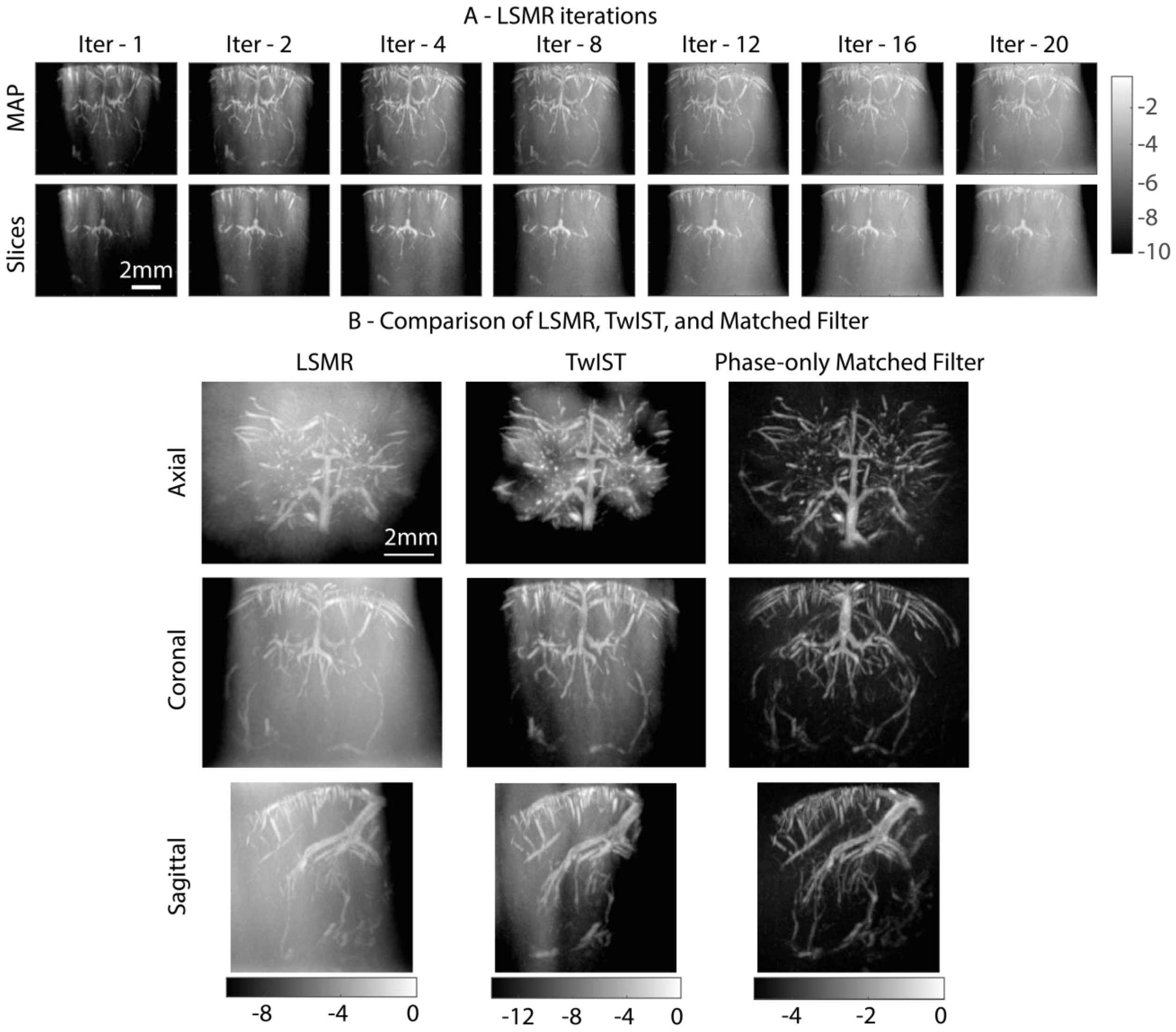

Fig. S7. Comparison of reconstruction methods for blood flow imaging.

(**A**) Maximum amplitude projections (top row) and slices (bottom row) through PDIs reconstructed with different iteration numbers of the LSMR algorithm. All of the volumes formed in the figure were formed by reconstructing the full 8041 frames comprising the anesthetized dataset, however, the data was filtered differently to the images presented in Fig. 2 and Fig. S3. We used only the 5501-6500 spatial singular vectors to increase the computational efficiency of the iterative methods. The slices were formed from maximum amplitude projections through an 800 µm coronal cross section. With increasing iteration number, the spatial variation in the amplitude diminishes at the expense of diminished contrast. (**B**) Axial, coronal, and sagittal maximum amplitude projections of PDIs formed with the three different reconstruction methods evaluated in this work. The LSMR projection was taken from iteration 16, the TwIST algorithm was first run to convergence followed by the use of the conjugate gradient method to debias the final volume.

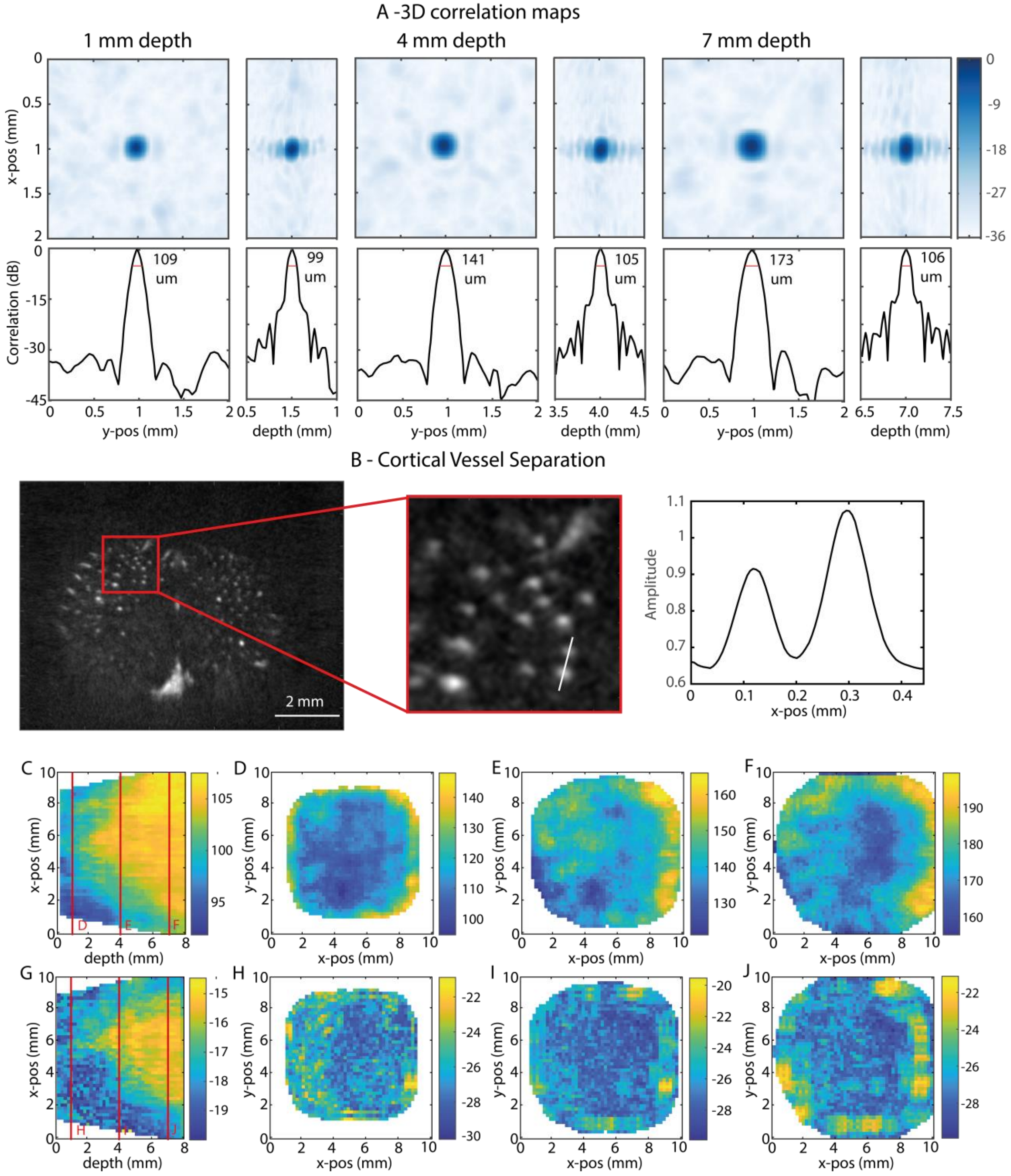

Fig. S8. Analysis of the spatial resolution of the imaging system.

(A) Analysis of the axial and lateral correlations of the matrix *A* with a fixed position (*x*,*y*) at 3 different depths. The correlations were evaluated on a grid with a 40 µm step size over a 2×2×1 mm volume centered on the voxel of interest. The top-row shows maximum amplitude projections through the spatial correlations

over this volume at each depth. The bottom row plots lateral and axial cross-sections through the point of interest for each depth. The values in each sub-figure of the bottom row denote the -6dB width. **(B)** Assessment of vessel resolution in the cortex approximately 0.6 *mm* inside the mouse brain. Separations of less than 200 µm can be identified supporting the assessment of the spatial resolution from the system matrix. **(C-F)** Variation in lateral resolution of system matrix on one axial cross-section (C) and lateral cross-sections at 1 (D), 4 (E), and (7) mm in depth. **(G-J)** Variation in side-lobe levels of system matrix on one axial cross-section (C) and lateral cross-sections at 1 (D), 4 (E), and (7) mm in depth. The resolution and sidelobes were evaluated with a 200 µm spacing in all cases.

Movie S1.

Axial flythrough of the Power Doppler Image shown in Fig. 2A. Projections taken through 0.24 mm thickness slices.

Movie S2.

Coronal and Sagittal flythrough of the Power Doppler Image shown in Fig. 2A. The top left figure shows a maximum amplitude projection through the axial view. The top right figure shows a moving projection through Sagittal slices with the current view being indicated by the moving green bar on the axial MAP. The bottom left figure shows a moving projection through Coronal slices with the current view being indicated by the moving white bar.

Movie S3.

Variation in reconstructed image quality with element number for a fixed probe aperture both covered and uncovered by an encoding mask. The mask was modelled as a thin-phase element that introduced a phase offset proportional to its thickness. We simulated RF acquisition as $u = Av$ and reconstructed using matched filter. For highly under-sampled probes the uncovered probe suffers from poor lateral resolution due to the directivity of its large elements as the sampling improves the reconstructed images of both methods converge toward one another.

Movie S4.

Maximum amplitude projections through the calibrated time domain scans of the forward field of each matrix element. (Top left) (x-y) projection, (top right) (y-t) projection, (bottom left) (x-t) projection.

REFERENCES AND NOTES

1. M. Tanter, M. Fink, Ultrafast imaging in biomedical ultrasound, *IEEE Trans. Ultrason. Ferroelectr. Freq. Control* **61**, 102–119 (2014.

2. M. Couade, The advent of ultrafast ultrasound in vascular imaging: A review, *J. Vasc. Diagn. Interv.* **4,** 9–22 (2016).

3. F. Sebag, J. Vaillant-Lombard, J. Berbis, V. Griset, J. F. Henry, P. Petit, C. Oliver, Shear wave elastography: A new ultrasound imaging mode for the differential diagnosis of benign and malignant thyroid nodules, *J. Clin. Endocrinol. Metabol.* **95**, 5281–5288 (2010).

4. J. Vappou, J. Luo, E. E. Konofagou, Pulse wave imaging for noninvasive and quantitative measurement of arterial stiffness in vivo, *Am. J. Hypertens.* **23**, 393–398 (2010).

5. C. Errico, J. Pierre, S. Pezet, Y. Desailly, Z. Lenkei, O. Couture, M. Tanter, Ultrafast ultrasound localization microscopy for deep super-resolution vascular imaging *Nature* **527**, 499 502 (2015).

6. K. Christensen-Jeffries, O. Couture, P. A. Dayton, Y. C. Eldar, K. Hynynen, F. Kiessling, M. O'Reilly, G. F. Pinton, G. Schmitz, M. X. Tang, M. Tanter, R. J. van Sloun, Super-resolution ultrasound imaging, *Ultrasound Med. Biol.* **46**, 865–891 (2020).

7. J. Bercoff, G. Montaldo, T. Loupas, D. Savery, F. Mézière, M. Fink, M. Tanter, Ultrafast compound doppler imaging: Providing full blood flow characterization. *IEEE Trans. Ultrason. Ferroelectr. Freq. Control* **58**, 134–147 (2011).

8. E. Mace, G. Montaldo, I. Cohen, M. Baulac, M. Fink, M. Tanter, Functional ultrasound imaging of the brain. *Nat. Methods* **8**, 662–664 (2011).

9. T. Deffieux, C. Demené, M. Tanter, Functional ultrasound imaging: A new imaging modality for neuroscience. *Neuroscience* **474**, 110–121 (2021).

10. N. Renaudin, C. Demené, A. Dizeux, N. Ialy-Radio, S. Pezet, M. Tanter, Functional ultrasound localization microscopy reveals brain-wide neurovascular activity on a microscopic scale. *Nat. Methods* **19**, 1004–1012 (2022).

11. J. Provost, C. Papadacci, J. E. Arango, M. Imbault, M. Fink, J.-L. Gennisson, M. Tanter, M. Pernot, 3D ultrafast ultrasound imaging in vivo. *Phys. Med. Biol.* **59**, L1–L13 (2014).

12. L. Petrusca, F. Varray, R. Souchon, A. Bernard, J.-Y. Chapelon, H. Liebgott, W. A. N'Djin, M. Viallon, A new high channels density ultrasound platform for advanced 4D cardiac imaging, in *Proceedings of the 2017 IEEE International Ultrasonics Symposium* (*IUS*), Washington, DC, USA, 6–9 September 2017, pp. 1–4.

13. A. Chavignon, B. Heiles, V. Hingot, C. Orset, D. Vivien, O. Couture, 3D transcranial ultrasound localization microscopy in the rat brain with a multiplexed matrix probe, *I.E.E.E. Trans. Biomed. Eng.* **69**, 2132–2142 (2022).

14. B. Heiles, M. Correia, V. Hingot, M. Pernot, J. Provost, M. Tanter, O. Couture, Ultrafast 3D ultrasound localization microscopy using a 32 × 32 matrix array. *IEEE Trans. Med. Imaging* **38**, 2005–2015 (2019).

15. C. Rabut, M. Correia, V. Finel, S. Pezet, M. Pernot, T. Deffieux, M. Tanter, 4D functional ultrasound imaging of whole-brain activity in rodents, *Nat. Methods* **16**, 994–997 (2019).

16. C. Chen, Z. Chen, D. Bera, S. B. Raghunathan, M. Shabanimotlagh, E. Noothout, Z.-Y. Chang, J. Ponte, C. Prins, H. J. Vos, J. G. Bosch, M. Verweij, N. de Jong, M. A. Pertijs, A front-end ASIC with receive sub-array beamforming integrated with a 32 × 32 PZT matrix transducer for 3-D transesophageal echocardiography. *IEEE J. Solid-State Circuits* **52**, 994–1006 (2017).

17. J. Janjic, M. Tan, V. Daeichin, E. Noothout, C. Chen, Z. Chen, Z.-Y. Chang, R. H. Beurskens, G. Van Soest, A. F. Van Der Steen, M. Verweij, M. A. Pertijs, N. de Jong, A 2-D ultrasound transducer with front-end ASIC and low cable count for 3-D forward-looking intravascular imaging: Performance and characterization. *IEEE Trans. Ultrason. Ferroelectr. Freq. Control* **65**, 1832–1844 (2018).

18. J. Sauvage, J. Porée, C. Rabut, G. Férin, M. Flesch, B. Rosinski, A. Nguyen-Dinh, M. Tanter, M. Pernot, T. Deffieux, 4D functional imaging of the rat brain using a large aperture row-column array. *IEEE Trans. Med. Imaging* **39**, 1884–1893 (2020).

19. L. Wei, G. Wahyulaksana, B. Meijlink, A. Ramalli, E. Noothout, M. D. Verweij, E. Boni, K. Kooiman, A. F. Van Der Steen, P. Tortoli, N. de Jong, H. J. Vox, High frame rate volumetric imaging of microbubbles using a sparse array and spatial coherence beamforming, *IEEE Trans. Ultrason. Ferroelectr. Freq. Control* **68**, 3069–3081 (2021).

20. A. Ramalli, E. Boni, E. Roux, H. Liebgott, P. Tortoli, Design, implementation, and medical applications of 2-D ultrasound sparse arrays, *IEEE Trans. Ultrason. Ferroelectr. Freq. Control,* **69** 2739–2755 (2022).

21. H. Favre, M. Pernot, M. Tanter, C. Papadacci, Transcranial 3D ultrasound localization microscopy using a large element matrix array with a multi-lens diffracting layer: An in vitro study, *Phys. Med. Biol.* **68**, 075003 (2023).

22. T. L. Szabo, *Diagnostic Ultrasound Imaging: Inside Out* (Academic Press, 2004).

23. P. Kruizinga, P. van der Meulen, A. Fedjajevs, F. Mastik, G. Springeling, N. de Jong, J. G. Bosch, G. Leus, Compressive 3D ultrasound imaging using a single sensor. *Sci. Adv.* **3**, e1701423 (2017).

24. N. Antipa, G. Kuo, R. Heckel, B. Mildenhall, E. Bostan, R. Ng, L. Waller, Diffusercam: Lensless single exposure 3D imaging. *Optica* **5**, 1 (2018).

25. J. D. Malone, N. Aggarwal, L. Waller, A. K. Bowden, Diffuserspec: Spectroscopy with scotch tape. *Opt. Lett.* **48**, 323–326 (2023).

26. A. C. Tondo Yoya, B. Fuchs, M. Davy, Computational passive imaging of thermal sources with a leaky chaotic cavity. *Appl. Phys. Lett.* **111**, 193501 (2017).

27. T. Fromenteze, O. Yurduseven, M. F. Imani, J. Gollub, C. Decroze, D. Carsenat, D. R. Smith, Computational imaging using a mode-mixing cavity at microwave frequencies. *Appl. Phys. Lett.* **106**, 194104 (2015).

28. M. P. Edgar, G. M. Gibson, M. J. Padgett, Principles and prospects for single-pixel imaging, *Nat. Photonics* **13**, 13–20 (2019).

29. J. N. Mait, G. W. Euliss, R. A. Athale, Computational imaging. *Adv. Opt. Photonics* **10**, 409 (2018).

30. C. Draeger, M. Fink, One-channel time-reversal in chaotic cavities: Theoretical limits. *J. Acoust. Soc. Am.* **105**, 611–617 (1999).

31. A. Derode, A. Tourin, M. Fink, Ultrasonic pulse compression with one-bit time reversal through multiple scattering, *J. Appl. Phys.* **85**, 6343–6352 (1999).

32. G. Montaldo, D. Palacio, M. Tanter, and M. Fink, Time reversal kaleidoscope: A smart transducer for threedimensional ultrasonic imaging. *Appl. Phys. Lett.* **84**, 3879–3881 (2004).

33. J. Robin, A. Simon, B. Arnal, M. Tanter, M. Pernot, Self-adaptive ultrasonic beam amplifiers: Application to transcostal shock wave therapy. *Phys. Med. Biol.* **63**, 175014 (2018).

34. O. Caron-Grenier, J. Poree, V. Perrot, G. Ramos Palacio, A. F. Sadikot, J. Provost, Ergodic encoding for single-element ultrasound imaging in vivo, arXiv:2308.09196 (2023).

35. Y. Li, L. Li, L. Zhu, K. Maslov, J. Shi, P. Hu, E. Bo, J. Yao, J. Liang, L. Wang, L. V. Wang, Snapshot photoacoustic topography through an ergodic relay for high-throughput imaging of optical absorption. *Nat. Photonics* **14**, 164–170 (2020).

36. Y. Li, T. T. Wong, J. Shi, H.-C. Hsu, L. V. Wang, Multifocal photoacoustic microscopy using a single-element ultrasonic transducer through an ergodic relay. *Light Sci. Appl.* **9**, 135 (2020).

37. L. Li, Y. Li, Y. Zhang, L. V. Wang, Snapshot photoacoustic topography through an ergodic relay of optical absorption in vivo. *Nat. Protoc.* **16**, 2381–2394 (2021).

38. J. Janjic, P. Kruizinga, P. van der Meulen, G. Springeling, F. Mastik, G. Leus, J. G. Bosch, A. F. van der Steen, G. van Soest, Structured ultrasound microscopy, *Appl. Phys. Lett.* **112**, 251901 (2018).

39. J. A. Jensen, S. I. Nikolov, K. L. Gammelmark, M. H. Pedersen, Synthetic aperture ultrasound imaging, *Ultrasonics* **44**, e5–e15 (2006), .

40. R. Y. Chiao, L. J. Thomas, S. D. Silverstein, Sparse array imaging with spatially-encoded transmits, in *1997 IEEE Ultrasonics Symposium Proceedings. An International Symposium* (*catalog no. 97CH36118*), vol. 2, Toronto, ON, Canada, 5–8 October 1997, pp. 1679–1682.

41. C. Demené, T. Deffieux, M. Pernot, B.-F. Osmanski, V. Biran, J.-L. Gennisson, L.-A. Sieu, A. Bergel, S. Franqui, J.-M. Correas, I. Cohen, O. Baud, M. Tanter, Spatiotemporal clutter filtering of

ultrafast ultrasound data highly increases Doppler and fultrasound sensitivity. *IEEE Trans. Med. Imaging* **34**, 2271–2285 (2015).

42. C. Madiena, J. Faurie, J. Porée, D. Garcia, Color and vector flow imaging in parallel ultrasound with sub-Nyquist sampling. *IEEE Trans. Ultrason. Ferroelectr. Freq. Control* **65**, 795–802 (2018).
43. H. Hu, H. Huang, M. Li, X. Gao, L. Yin, R. Qi, R. S. Wu, X. Chen, Y. Ma, K. Shi, C. Li, T. M. Maus, B. Huang, C. Lu, M. Lin, S. Zhou, Z. Lou, Y. Gu, Y. Chen, Y. Lei, X. Wang, R. Wang, W. Yue, X. Yang, Y. Bian, J. Mu, G. Park, S. Xiang, S. Cai, P. W. Corey, J. Wang, S. Xu, A wearable cardiac ultrasound imager, *Nature* **613**, 667–675 (2023).
44. C. Demen'e, J. Robin, A. Dizeux, B. Heiles, M. Pernot, M. Tanter, and F. Perren, Transcranial ultrafast ultrasound localization microscopy of brain vasculature in patients, *Nat. Biomed. Eng.* **5**, 219–228 (2021).
45. G. Arya, W. F. Li, C. Roques-Carmes, M. Soljačić, S. G. Johnson, Z. Lin, End-to-end optimization of metasurfaces for imaging with compressed sensing. arXiv:2201.12348 (2022).
46. M. R. Kellman, E. Bostan, N. A. Repina, L. Waller, Physics-based learned design: Optimized coded-illumination for quantitative phase imaging. *IEEE Trans. Comput. Imaging* **5**, 344–353 (2019).
47. E. Markley, F. L. Liu, M. Kellman, N. Antipa, L. Waller, Physics-based learned diffuser for single-shot 3D imaging, in *NeurIPS 2021 Workshop on Deep Learning and Inverse Problems* (NeurIPS, 2021).
48. K. Melde, A. G. Mark, T. Qiu, and P. Fischer, Holograms for acoustics, *Nature* **537**, 518–522 (2016).
49. J. Lopes, M. Andrade, J. Leao-Neto, J. Adamowski, I. Minin, G. Silva, Focusing acoustic beams with a ball-shaped lens beyond the diffraction limit. *Phys. Rev. Appl.* **8**, 024013 (2017).
50. C. Holmes, B. W. Drinkwater, P. D. Wilcox, Postprocessing of the full matrix of ultrasonic transmit–receive array data for non-destructive evaluation. *NDT E Int.* **38**, 701–711 (2005).
51. A. Bertolo, M. Nouhoum, S. Cazzanelli, J. Ferrier, J. C. Mariani, A. Kliewer, B. Belliard, B.-F. Osmanski, T. Deffieux, S. Pezet, Z. Lenkei, M. Tanter, Whole-brain 3D activation and functional

connectivity mapping in mice using transcranial functional ultrasound imaging, *J. Vis. Exp.* e62267 (2021).

52. P. Kaczkowski, Bandwidth sampling data acquisition with the vantage system for high frequency transducers Verasonics, Kirkland, WA, USA, White Paper, 2016, pp. 1–5.

53. M.-H. Bae, W.-Y. Lee, M.-K. Jeong, S.-J. Kwon, Orthogonal golay code based ultrasonic imaging without reducing frame rate, in *Proceedings of the 2002 IEEE Ultrasonics Symposium*, Vol. 2, Munich, Germany, 8–7 October 2002, pp. 1705–1708.

54. P. van der Meulen, P. Kruizinga, J. G. Bosch, G. Leus, Calibration techniques for single-sensor ultrasound imaging with a coding mask, in *Proceedings of the 2018 52nd Asilomar Conference on Signals, Systems, and Computers*, Pacific Grove, CA, USA, 28–31 October 2018, pp. 1641–1645.

55. K. A. Wear, Spatiotemporal deconvolution of hydrophone response for linear and nonlinear beams—Part I: Theory, spatial-averaging correction formulas, and criteria for sensitive element size. *IEEE Trans. Ultrason. Ferroelectr. Freq. Control* **69**, 1243–1256 (2022).

56. X. Zeng, R. J. McGough, Evaluation of the angular spectrum approach for simulations of near-field pressures. *J. Acoust. Soc. Am.* **123**, 68–76 (2008).

57. D. C.-L. Fong, M. Saunders, LSMR: An iterative algorithm for sparse least-squares problems. *SIAM J. Sci. Comput. Secur.* **33**, 2950–2971 (2011).

58. C. C. Paige, M. A. Saunders, LSQR: An algorithm for sparse linear equations and sparse least squares, *ACM Trans. Math. Softw.* **8**, 43–71 (1982).

59. B. Berthon, P. Morichau-Beauchant, J. Porée, A. Garofalakis, B. Tavitian, M. Tanter, J. Provost, Spatiotemporal matrix image formation for programmable ultrasound scanners, *Phys. Med. Biol.* **63**, 03NT03 (2018).

60. J. M. Bioucas-Dias, M. A. Figueiredo, A new twist: Two-step iterative shrinkage/thresholding algorithms for image restoration, *IEEE Trans. Image Process.* **16**, 2992–3004 (2007).

61. M. A. Figueiredo, R. D. Nowak, S. J. Wright, Gradient projection for sparse reconstruction: Application to compressed sensing and other inverse problems. *IEEE J. Sel. Top. Signal Process.* **1**, 586–597 (2007).

62. D. Dogan, P. Kruizinga, J. G. Bosch, G. Leus, Multiple measurement vector model for sparsity-based vascular ultrasound imaging, in *Proceedings of the 2021 IEEE Statistical Signal Processing Workshop* (*SSP*), Rio de Janeiro, Brazil, 11–14 July 2021, pp. 501–505.

63. A. Bar-Zion, O. Solomon, C. Tremblay-Darveau, D. Adam, Y. C. Eldar, Sushi: Sparsity-based ultrasound super-resolution hemodynamic imaging. *IEEE Trans. Ultrason. Ferroelectr. Freq. Control* **65**, 2365–2380 (2018).

64. H. Estrada, J. Rebling, J. Turner, D. Razansky, Broadband acoustic properties of a murine skull. *Phys. Med. Biol.* **61**, 1932–1946 (2016).

65. B. E. Treeby, B. T. Cox, k-Wave: MATLAB toolbox for the simulation and reconstruction of photoacoustic wave fields. *J. Biomed. Opt.* **15**, 021314 (2010).

66. F. Bureau, J. Robin, A. Le Ber, W. Lambert, M. Fink, A. Aubry, Three-dimensional ultrasound matrix imaging. *Nat. Commun.* 14, 6793 (2023).

67. P. van der Meulen, M. Coutiño, J. G. Bosch, P. Kruizinga, G. Leus, Ultrasonic imaging through aberrating layers using covariance matching. *IEEE Trans. Comput. Imaging* **9**, 745–759 (2023).
